# Single‐Stage Fixation of Ipsilateral Tibial Plateau and Tibial Plafond Fractures: A Case Report

**DOI:** 10.1155/cro/8037873

**Published:** 2026-09-15

**Authors:** Tuan Anh Nguyen, Man Duc Minh Phan, Phi Duong Nguyen

**Affiliations:** ^1^ Department of Orthopaedics, Pham Ngoc Thach University of Medicine, Ho Chi Minh City, Vietnam, pnt.edu.vn; ^2^ Lower Limb Surgery Department, Hospital for Traumatology and Orthopaedics, Ho Chi Minh City, Vietnam; ^3^ Orthopaedic-Burn-Plastic Surgery Department, City Children′s Hospital, Ho Chi Minh City, Vietnam

**Keywords:** case report, minimally invasive plate osteosynthesis, pilon fracture, Schatzker VI fracture, tibial plateau fracture

## Abstract

**Background:**

Simultaneous ipsilateral fractures of the proximal and distal tibial articular ends are uncommon. Although this pattern has sometimes been described as a ‘floating tibia’, the term is not standardised. Management is dictated by fracture morphology, soft‐tissue status, patient physiology and the ability to obtain stable articular reduction without additional soft‐tissue injury.

**Case Presentation:**

A 45‐year‐old man sustained a closed Schatzker VI tibial plateau fracture, an ipsilateral partial‐articular distal tibial plafond fracture documented as AO/OTA 43‐B2 and a comminuted fibular fracture after a motorcycle accident. The patient was haemodynamically stable, had intact distal neurovascular findings and had marked but clinically viable soft tissues without open wounds or fracture blisters (Tscherne Grade II). Preoperative CT was performed and informed planning; however, the original CT data and reconstructed images are no longer retrievable because of limited long‐term digital archiving and storage resources in the treating setting in Vietnam. Single‐stage fixation was undertaken within 10 h of injury after repeated clinical assessment. The plateau was treated with limited open elevation of the depressed lateral articular surface and submuscular lateral locking‐plate fixation; the fibula was plated, and the distal plafond split was reduced percutaneously and fixed with cannulated lag screws. At 6 months, plain radiographs demonstrated union without loss of alignment (MPTA 87°, LDTA 89°). Knee motion was 0°–120°; ankle motion was documented as 10° dorsiflexion and 30° plantarflexion. The Knee Society scores were 85 (knee) and 80 (function), and the AOFAS Ankle‐Hindfoot Scale score was 82. No wound infection, loss of reduction, implant failure, nonunion or unplanned reoperation was documented in the available follow‐up record. Elective implant removal was performed at 18 months after union at the patient′s request.

**Conclusion:**

Single‐stage fixation was technically feasible in this highly selected patient with stable physiology, a closed injury, a clinically viable soft‐tissue envelope and a distal articular fracture. This single case does not establish equivalence or superiority to staged management, particularly for severely comminuted or soft‐tissue‐compromised pilon injuries.

## 1. Introduction

Simultaneous ipsilateral fractures affecting both the tibial plateau and the distal tibial plafond are rare. The expression floating tibia has been used descriptively for noncontiguous fractures of the proximal and distal tibia with an intervening diaphyseal segment, but unlike ‘floating knee’ it is not a uniformly defined or widely indexed diagnostic term. For clarity, this report therefore uses the anatomical description ‘ipsilateral tibial plateau and tibial plafond fractures’. A focused, non‐systematic literature search performed for this revision identified very limited directly comparable material; notably, one patient with an ipsilateral tibial plateau and pilon fracture was included in a 2023 technical case series of periarticular fracture reduction [1]. Most available evidence addresses high‐energy plateau and pilon fractures separately.

Treatment principles for both injuries emphasise restoration of articular congruity and limb alignment while protecting the soft‐tissue envelope. Staged protocols are widely used for high‐energy pilon fractures and high‐energy proximal tibial fractures when swelling, blistering, open injury, severe comminution, contamination, physiological instability or uncertainty about soft‐tissue viability is present [2, 3]. However, observational studies have also reported acceptable outcomes after acute definitive fixation in selected patients treated by experienced trauma teams [4, 5]. These data support individualised timing rather than a fixed ‘golden period’.

The present report describes single‐stage treatment of a Schatzker VI plateau injury together with a partial‐articular distal tibial plafond fracture. The educational focus is the selection process, the operative sequence and the precautions used to limit additional soft‐tissue injury rather than a claim that single‐stage fixation is preferable to staged management. This report follows the CARE guideline framework [6].

## 2. Case Presentation

### 2.1. Patient Information and Initial Clinical Findings

A 45‐year‐old man presented approximately 2 h after a motorcycle accident with severe pain, deformity, shortening and marked swelling of the right lower leg. He had no significant recorded medical history, was a non‐smoker, and was haemodynamically stable. The injury was closed. Distal pulses were palpable, capillary refill was normal and distal sensation was intact.

The soft‐tissue envelope was markedly swollen but had no open wound, fracture blister, bulla or skin‐tension tear. The injury was documented as Tscherne Grade II [7]. A formal preoperative wrinkle‐sign assessment was not recorded in the available chart. Repeated clinical examinations assessed pain, pain on passive stretch, compartment firmness, distal perfusion and neurological status. No evolving clinical features of compartment syndrome were documented. No invasive compartment‐pressure measurements are recorded in the source chart; therefore, no numerical pressure or delta‐pressure values are reported. The clinical course is presented chronologically in Table 1.

**Table 1:** Clinical timeline.

Time point	Clinical event
Injury	Motorcycle accident; high‐energy mechanism.
~2 h after injury	Arrival at hospital; haemodynamically stable; closed injury; marked swelling; intact distal neurovascular examination.
Preoperative period	Plain radiographs and CT obtained. Serial clinical soft‐tissue and compartment assessments performed; no evolving clinical compartment syndrome documented.
Within 10 h of injury	Decision for definitive fixation based on stable physiology, closed injury, clinically viable soft tissues, absence of neurovascular compromise, reconstructible fracture morphology and availability of an experienced trauma team.
Operation	Single‐stage fixation: limited open reduction/submuscular lateral plate fixation of plateau, fibular plating and percutaneous lag‐screw fixation of distal tibial plafond. Total operative time 95 min; estimated blood loss 150 mL.
Postoperative Day 3	Passive and active‐assisted knee and ankle range‐of‐motion exercises initiated.
Postoperative Day 7	Discharged from hospital.
0–8 weeks	Nonweight‐bearing.
After early radiographic union	Progression to partial weight‐bearing.
~12–14 weeks	Full weight‐bearing permitted.
6 months	Radiographic union and alignment assessment; knee and ankle range of motion and functional scores recorded.
18 months	Complete union confirmed on plain radiographs; elective implant removal performed at the patient′s request; no additional clinical indication for removal was documented in the available record.

### 2.2. Diagnostic Assessment

**Figure 1:**
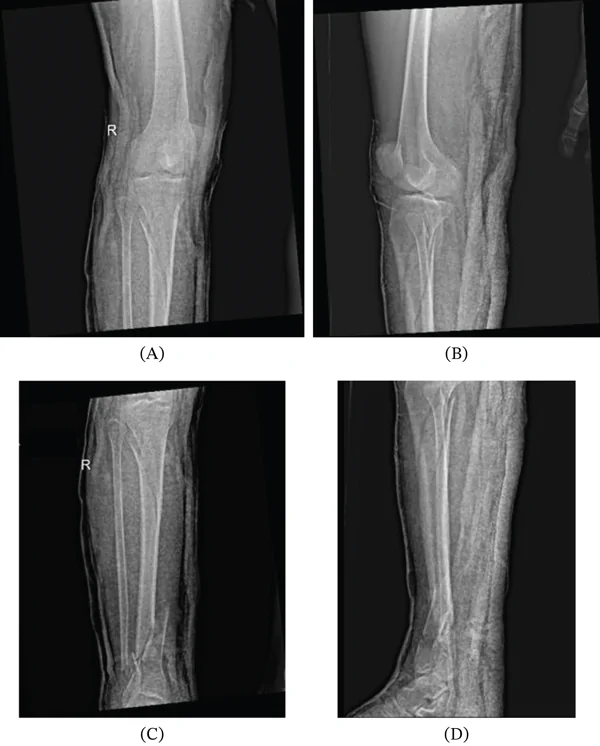
Preoperative plain radiographs. (A) Anteroposterior knee view and (B) lateral knee view demonstrate the bicondylar proximal tibial injury. (C) Anteroposterior distal‐leg/ankle view and (D) lateral distal‐leg/ankle view demonstrate the distal tibial plafond and associated fibular fractures. CT was obtained preoperatively but the original CT image files are no longer retrievable because of archival and storage limitations described in the text.

Plain radiographs demonstrated a bicondylar tibial plateau fracture, a distal tibial articular fracture and a comminuted fibular fracture (Figure 1). Preoperative CT of the knee and ankle was performed for surgical planning. The contemporaneous CT description documented a Schatzker VI bicondylar split‐depression fracture with approximately 8‐mm depression of the lateral plateau and a coronal split of the medial plateau. The distal tibial injury was documented as an AO/OTA 43‐B2 partial‐articular sagittal split involving the weight‐bearing plafond, with < 2‐mm articular displacement, an intact posterior malleolar fragment, no metaphyseal comminution and no talar subluxation. Because the distal lesion involved the tibial plafond, the terms ‘pilon’ and ‘plafond fracture’ are used descriptively, while acknowledging that its morphology was substantially less comminuted than a classic high‐energy AO/OTA 43‐C pilon injury.

The original CT DICOM files and derived axial, coronal, sagittal and three‐dimensional reconstructions are no longer retrievable from the hospital archive. According to the treating team, this reflects limited long‐term digital imaging storage and archival resources in the local resource‐constrained setting in Vietnam. Consequently, CT images cannot be supplied with this revision. The CT morphology reported above is based on the contemporaneous documented interpretation rather than retrospective re‐reading. This is an important limitation of the report. The proximal injury was contemporaneously classified as Schatzker VI; an additional AO/OTA subtype was not assigned retrospectively because the original CT images are unavailable for reliable reclassification.

### 2.3. Therapeutic Intervention

The decision to proceed with single‐stage fixation was individualised. It was not based on an arbitrary < 12‐hour golden period. The principal considerations documented were stable haemodynamics, a closed injury, intact distal neurovascular status, absence of clinically evolving compartment syndrome, no fracture blisters or skin breakdown, a distal fracture pattern amenable to percutaneous fixation and the availability of an experienced trauma team. Staged temporary external fixation would have remained appropriate if the soft tissues had deteriorated or if fracture morphology had required more extensive exposure.

Surgery was performed under spinal anaesthesia with the patient supine on a radiolucent table. Total operative time was 95 min and estimated blood loss was 150 mL.

**Figure 2:**
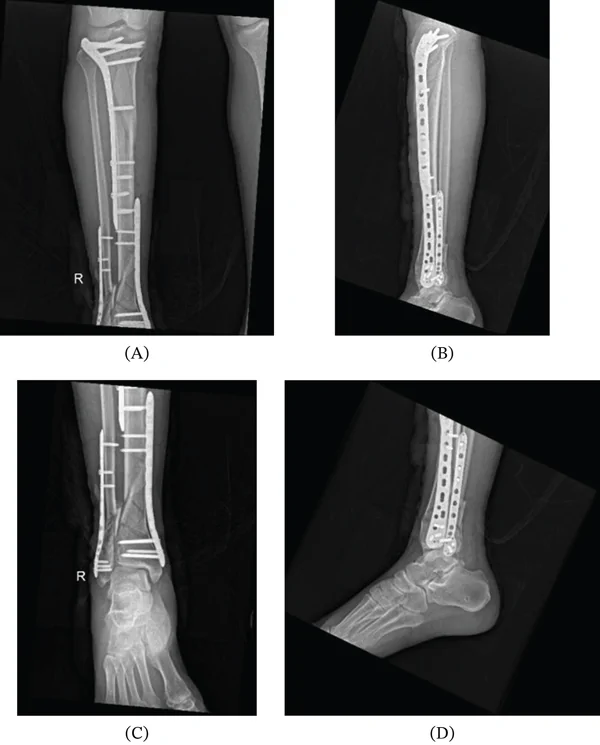
Postoperative radiographs. (A) Anteroposterior and (B) lateral views of the tibia demonstrate lateral locked‐plate fixation of the proximal tibia and plate fixation of the fibula. (C) Anteroposterior and (D) lateral ankle views demonstrate percutaneous screw fixation of the distal tibial plafond component and restoration of coronal and sagittal alignment. Immediate postoperative plain‐radiograph measurements were MPTA 87°, LDTA 89°, and posterior distal tibial angle 78°. Residual articular incongruity was estimated as < 1 mm on the available intraoperative/postoperative radiographic assessment; no postoperative CT is available for confirmation.

1. Step 1 proximal tibia: Through a limited anterolateral window, the depressed lateral articular surface was directly elevated with a bone tamp and supported with cancellous bone chips. The source of the cancellous chips was not specified in the archived operative record. A precontoured 4.5‐mm lateral locking compression plate was inserted submuscularly, and rafting screws supported the lateral articular surface. This is more accurately described as limited open articular reduction combined with minimally invasive plate insertion rather than a purely indirect MIPO reduction. No separate posteromedial plate was used. The archived documentation does not provide sufficient detail to reconstruct the exact reduction manoeuvre for the medial coronal component or the case‐specific reason a supplementary medial buttress plate was not used; this limitation is acknowledged and the construct is shown in Figure 2.
2. Step 2–fibula: Through a limited distal‐lateral approach, the fibular shaft fracture was reduced and stabilised with a 3.5‐mm locking compression plate. Fibular length was restored before definitive distal tibial fixation to assist restoration of the lateral column and distal tibial alignment. In this patient, this sequence did not prevent subsequent percutaneous reduction of the distal articular split.
3. Step 3–distal tibial plafond: Traction restored length and alignment. The sagittal articular fragment was manipulated percutaneously under biplanar fluoroscopy, including use of a 3‐mm joystick pin, and fixed with three 4.0‐mm cannulated lag screws from anterolateral to posteromedial. The previous description of a ‘major die‐punch fragment’ has been removed because it was inconsistent with the documented CT morphology of a simple sagittal partial‐articular split. The use of minimally invasive surgical techniques at the distal tibia to minimize additional soft‐tissue injury and promote fracture healing

The available archived operative documentation did not preserve tourniquet duration, fluoroscopy time, implant manufacturer, exact graft provenance, drain use or detailed wound‐closure technique. These details have therefore not been reconstructed. No postoperative wound‐healing problem is documented in the available follow‐up record.

### 2.4. Postoperative Care and Follow‐Up

Passive and active‐assisted motion began on postoperative Day 3 and progressed as tolerated. The patient remained nonweight‐bearing for 8 weeks, progressed to partial weight‐bearing after early radiographic evidence of healing and was permitted full weight‐bearing at approximately 12–14 weeks. The patient was discharged on postoperative Day 7. The exact pharmacological and/or mechanical venous‐thromboembolism prophylaxis regimen was not retrievable from the archived record and is therefore not reported. Outcomes and adverse‐event surveillance are summarized in Table 2.

**Table 2:** Outcomes and adverse‐event surveillance.

Time point	Function/weight‐bearing	Scores	Radiographic findings	Documented adverse events/procedures
Immediate postoperative	Protected mobilisation	Not formally scored	Alignment restored; MPTA 87°, LDTA 89°, posterior distal tibial angle 78°; residual incongruity estimated < 1 mm on radiographic assessment	No immediate neurovascular deterioration or clinical compartment syndrome documented.
6 months	Full weight‐bearing achieved by ~12–14 weeks; knee ROM 0°–120°; ankle dorsiflexion 10°, plantarflexion 30°	Knee Society: 85 knee/80 function; AOFAS Ankle‐Hindfoot: 82	Union at proximal tibia, distal tibia and fibula; maintained alignment	Available record did not document wound breakdown/infection, clinically diagnosed VTE, loss of reduction, implant failure, delayed union, nonunion or unplanned reoperation.
18 months	Clinical follow‐up after union	Not formally rescored	Complete union and maintained alignment	Elective implant removal at patient request; no other reoperation documented.

For this report, radiographic union was judged on serial orthogonal plain radiographs by bridging trabecular/cortical continuity across the fracture regions, absence of progressive displacement and maintenance of alignment during progression of weight‐bearing. No postoperative CT was available. The alignment and articular measurements reported here were obtained from preserved plain radiographs; no formal blinded multiobserver reliability analysis was performed.

At 6 months, plain radiographs demonstrated union of the proximal tibia, distal tibia and fibula without loss of alignment. MPTA was 87° and LDTA was 89°. Knee range of motion was 0°–120°, which was considered functionally satisfactory but is not described as ‘full’ motion. Ankle range of motion was documented as 10° dorsiflexion and 30° plantarflexion. The 1989 Knee Society Clinical Rating System yielded scores of 85 for the knee and 80 for function [8]. The AOFAS Ankle‐Hindfoot Scale score was 82 [9]; because this instrument combines patient‐ and clinician‐derived components, it is not presented as a purely patient‐reported outcome measure.

**Figure 3:**
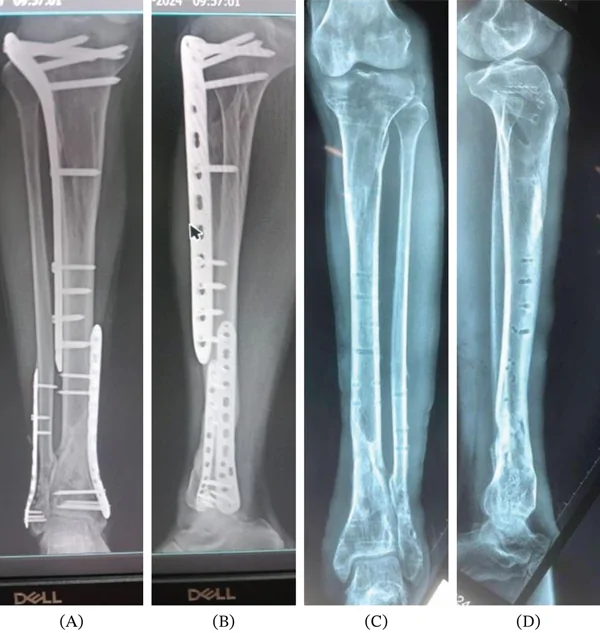
Eighteen‐month radiographs before and after elective implant removal. (A–B) Anteroposterior and lateral views before removal demonstrate maintained alignment and complete union. (C–D) Corresponding views after implant removal demonstrate preserved alignment and healed fracture sites. No image enhancement other than routine sizing and panel labelling was applied for this revision.

At 18 months, follow‐up radiographs showed complete union and maintained alignment. Implant removal was performed electively at the patient′s request after union. No additional mandatory clinical indication for removal was documented in the available record (Figure 3).

### 2.5. Patient Perspective

A formal contemporaneous CARE‐style patient narrative was not recorded. At the 6‐month review, the patient reported no significant pain or discomfort, and at 18 months he requested elective implant removal after fracture union. Return‐to‐work status, recreational activity, preinjury functional scores and a structured satisfaction assessment were not documented sufficiently to permit reliable retrospective reconstruction. We therefore report only the available patient‐reported information and acknowledge the absence of a fuller patient perspective as a limitation.

## 3. Discussion

The principal feature of this case is the coexistence of a complex bicondylar proximal tibial injury with a relatively simple partial‐articular distal plafond fracture. That distinction is central to interpretation. The favourable short‐ to medium‐term course cannot be attributed to ‘single‐stage surgery’ alone and should not be extrapolated to markedly comminuted AO/OTA 43‐C pilon fractures. The limited displacement and absence of metaphyseal comminution in the distal component made percutaneous lag‐screw fixation possible with relatively little additional soft‐tissue exposure. In cases with metaphyseal comminution, minimally invasive surgical techniques should be considered to limit soft‐tissue dissection.

The optimal timing of definitive fixation for high‐energy periarticular tibial fractures remains controversial. Staged management is well‐supported when the soft‐tissue envelope is compromised. Sirkin et al. established a staged soft‐tissue protocol for complex pilon fractures [2], and Egol et al. reported a standardised staged approach for high‐energy proximal tibial fractures [3]. Conversely, White et al. [4] and more recent comparative data from Flanagan et al. [5] show that acute definitive fixation can be acceptable in carefully selected patients treated by experienced orthopaedic trauma surgeons. These studies do not validate a universal time threshold. The key variables are soft‐tissue viability, physiological stability, fracture morphology, contamination/open injury, surgeon expertise and the ability to obtain stable fixation without excessive additional tissue injury.

The preoperative soft tissues in this case were not normal: marked swelling and Tscherne Grade II injury were present. The decision for early definitive treatment therefore depended on the absence of blistering, open wounds, neurovascular compromise or clinical deterioration during serial examinations, together with a reconstructible distal fracture pattern and a minimally invasive operative plan. A formal wrinkle sign was not recorded, and no invasive compartment‐pressure measurements were documented. These omissions limit retrospective certainty and are now stated explicitly rather than supplemented with unverified numerical values.

The proximal construct also warrants caution. High‐energy bicondylar plateau fractures frequently require more than one fixation column, and dual‐incision/dual‐plate strategies are established for selected AO/OTA 41‐C patterns [10]. In this patient, the preserved postoperative radiographs show a lateral locked plate without a separate posteromedial buttress plate. Because the original CT is unavailable and the operative note does not fully reconstruct the medial‐fragment reduction strategy, this case should not be interpreted as evidence that a lateral‐only construct is generally sufficient for Schatzker VI fractures.

Long‐term pilon outcomes depend not only on union but also on the quality of articular reconstruction and the degree of initial injury. In a 94‐fracture series with a mean follow‐up of approximately 56 months, Biz et al. reported substantial early and/or late complication burden and showed that clinical results were closely related to soft‐tissue condition and anatomic joint reconstruction [11]. These data reinforce why the present 18‐month follow‐up is insufficient to exclude later post‐traumatic arthritis. Similarly, recent plateau literature emphasises that bicondylar fracture severity is associated with slower and less complete functional recovery than lower‐severity patterns [12].

Primary ankle arthrodesis has a role in selected nonreconstructible, severely comminuted pilon injuries, but it was not appropriate in this patient because the distal articular surface was reconstructible and only minimally displaced. A systematic review found potentially reasonable results for primary arthrodesis but highlighted the limited quality and quantity of evidence [13]. Minimally invasive tibiotalocalcaneal arthrodesis with a retrograde nail has also been described [14], but this is a fusion/salvage strategy and should not be cited as direct support for percutaneous fixation of an acute AO/OTA 43‐B2 fracture.

The case also illustrates an important reporting problem in resource‐limited environments. CT was obtained and used for planning, but the original images were not retained in a retrievable long‐term digital archive. This prevents independent verification of the plateau morphology, precise AO/OTA subclassification and the distal articular description. The preserved plain radiographs still demonstrate the overall fracture configuration and fixation, but the absence of CT images materially limits the educational completeness of the case.

### 3.1. Limitations

This report has several limitations: it describes a single highly selected patient without a comparator; the distal fracture had favourable partial‐articular morphology and limited comminution; the original CT images are no longer retrievable; follow‐up of 18 months is too short to assess post‐traumatic arthritis reliably; preinjury functional data and a validated patient‐reported outcome measure were not available; the AOFAS and Knee Society scores include clinician‐assessed components and were not independently verified by multiple observers; radiographic measurements were based on preserved plain radiographs without postoperative CT; several operative details and the exact VTE prophylaxis regimen were not recoverable from the archived chart; a complete formal patient‐perspective narrative was not recorded; and the effects of operative timing cannot be separated from fracture morphology, minimally invasive technique, patient characteristics or surgeon expertise. These limitations substantially restrict generalisability.

## 4. Conclusion

Single‐stage fixation produced satisfactory radiographic and functional results in this specific patient, who was physiologically stable, had a closed injury with a clinically viable though moderately injured soft‐tissue envelope, intact neurovascular status and a relatively simple partial‐articular distal tibial plafond fracture. The case demonstrates technical feasibility under highly selected conditions, not equivalence or superiority to staged management. The main educational value is the explicit selection rationale, recognition of the different severities of the proximal and distal components, a soft‐tissue‐preserving operative sequence and careful acknowledgement of the information that cannot be verified retrospectively.

## Author Contributions

**Tuan Anh Nguyen**: conceptualization, data curation, investigation, writing—original draft. **Man Duc Minh Phan**: investigation, methodology, supervision, writing—review and editing. **Phi Duong Nguyen**: conceptualization, supervision, validation, writing—review and editing, final approval.

## Funding

No funding was received for this manuscript.

## Ethics Statement

Formal ethical approval was waived for this single‐patient case report in accordance with the treating institution′s policy because the report describes routine clinical care without an experimental intervention. No separate approval number was available in the archived case record. This case report was revised in accordance with the CARE reporting guidelines; a completed CARE checklist accompanies the revision.

## Consent

Written informed consent for publication of the case and de‐identified images was obtained from the patient. Written informed consent was obtained from the patient for publication of this case report and the accompanying de‐identified radiographs.

## Conflicts of Interest

The authors declare no conflicts of interest.

## Data Availability

The data that support the findings of this study are available from the corresponding author upon reasonable request.
